# Charles Blagden's diary: Information management and British science in the eighteenth century

**DOI:** 10.1098/rsnr.2018.0016

**Published:** 2018-05-23

**Authors:** Hannah Wills

**Affiliations:** Department of Science and Technology Studies, University College London, Gower Street, London WC1E 6BT, UK

**Keywords:** Royal Society, Joseph Banks, Charles Blagden, information management, patronage

## Abstract

This paper examines the diary of Charles Blagden, physician and secretary of the Royal Society between 1784 and 1797. It argues that the form and content of Blagden's diary developed in response to manuscript genres from a variety of contexts, including the medical training that Blagden undertook at the start of his career, the genre of the commonplace book, and contemporary travel narratives. Blagden was interested in the workings of memory and in the association of ideas. This paper reveals the diary's nature as an aid to memory and an information management tool. It argues that the diary assisted Blagden's attempts to secure the patronage of key figures in the eighteenth-century scientific world, including Joseph Banks, the Royal Society and a London-based network of aristocratic women. In exploring the development of the diary, the paper uncovers the role of a material object in aiding the management of patronage relationships central to the career of a significant but little-studied secretary of the Royal Society.

## Introduction

On 1 January 1795 Charles Blagden recorded the following in his diary:

Jan 1, 1795. [Thursday]. B[reakfaste]d at Sir J[oseph]. B[ank]s’. talked about Translation that doubted whether worth publishing. Gave Major Rennel character of Mr Wilberforce, harsh as he deserved. Called on L[or]d Lucan; began really to think something: shewed drawings of Sir W[illiam]. H[amilton]'s paper on Vesuvius: agreed about L[ad]y Spencer; L[or]d Bulkely there, friendlier; L[or]d Cambden poor weak character: executed very ill his speech. Then called on Montagus; very civil asked & offered about correcting arguments. Mr & Mrs Paradise called. Dined at the club; having first called on Mr Cavendish, & gave him translation of Fr[iar]. Breislak on Vesuvian Eruption to look over. Cold day: froze hard in night, had been on Soho Square 19°, at Clapham 11°, wind N[orth] S[outh] varying. fog at times & partially, not so much close to river, or N[orth]. part of town but between: froze about as much as at Bedford Square.^1^

This is a typical entry in the diary of Charles Blagden, physician and secretary of the Royal Society between 1784 and 1797. The diary is vast, spanning more than 40 years of its author's life, and in its terse and seemingly uninformative prose, alongside its difficult handwriting, has generally been viewed as something of a puzzle. Eight volumes of Blagden's diary, covering the years 1771 to 1820, are held within the Royal Society's collections, and were purchased alongside some of his letters, papers, and printed books in 1947.^2^ Additional material was presented to the Society in 2000, including a further 45 leaves of loose diary sections covering 1792 to 1794.^3^ Further volumes of the diary from the period 1776 to 1788 are held within the collections of the Beinecke Rare Book and Manuscript Library at Yale University, and comprise part of the Osborn Collection of English historical manuscripts, alongside a collection of Blagden's correspondence and papers.^4^ The diary consists of individual sheets folded in half, bound in small booklets of a few pages, with small cursive handwriting on the recto and verso.

Existing scholarship on Blagden's diary has primarily focused on brief excerpts as evidence of particular historical moments. Danielle Fauque has explored Blagden's diary and correspondence in order to trace exchanges between British and French scientists in the 1780s, focusing on the 1783 ‘water controversy’.^5^ Iain Watts has examined the diary from the period 1806 to 1814, exploring information exchanges concerning electrochemistry during Napoleon's Continental Blockade.^6^ In contrast, this paper considers the diary on its own terms, as a multi-faceted document for the management of information, built up through the use of diverse manuscript genres of record-keeping over Blagden's lifetime. ‘Information’, as Blagden used the term, covered all manner of news, ideas and observations, shared in conversation and correspondence. It included subjects ranging from the weather to politics, all recorded in the diary.^7^ This paper explores the diary as a tool of memory, self-fashioning and patronage, which assisted Blagden's attempts to pursue social prestige. If the diary seems terse and uninformative, this is not a reason to pass over it, but instead presents a historical characteristic that requires explanation. This paper attempts such an explanation.

Diaries have received increased attention in recent years in the history of early modern science. Michael Hunter and Charles Littleton have examined what they have termed Robert Boyle's ‘work-diary’, a manuscript containing notes on experiments, travellers’ testimonies and extracts from reading, collated throughout Boyle's career.^8^ Felicity Henderson has explored the nature of Robert Hooke's memoranda, often referred to as his diary, compiled to keep track of a range of information including news, household affairs and Hooke's employment at the Royal Society.^9^ As yet, no similar investigation of Blagden's diary has been conducted. Much remains to be explored of his approach to information management, and his changing careers as a medical practitioner, assistant and advisor to better-known figures such as Joseph Banks, as Royal Society secretary, and as a client of aristocratic London society. Building upon this literature, I argue that Blagden's diary was shaped by note-taking methods derived from medical practice, commonplace books and travel writing, alongside correspondence. Underlying Blagden's use of these genres were the ways in which the diary assisted him in pursuing careers in the service of advancement through patronage. Though his diary was not solely responsible for shaping his careers and relationships, this paper suggests that it nonetheless played a significant role.

The paper begins by considering Blagden's interest in memory and the association of ideas, exploring its influence on his habitual note-taking. There follows an examination of the chronological development of Blagden's career and note-taking, first as a student and physician in the 1760s and early 1770s, during which he compiled medical notes and a commonplace book, in the service of pursuing fame and fortune through a medical career. His brief service as surgeon to the army in North America in the mid 1770s is considered next, revealing how voyage narratives, alongside his desire to fashion a career as Banks's client, shaped the form of his diary. The final sections examine Blagden's career as Royal Society secretary, and his later attempts to foster a network of predominantly female aristocratic patrons, aided by the diary.

## The association of ideas and Blagden's interest in memory

In form, structure, and content, Blagden's diary and notebooks bear the influence of information management strategies derived from a number of manuscript genres from personal and professional contexts. What was common to each of these was the way in which they operated as *aides-mémoires*. In his student medical notes, compiled during his time at the University of Edinburgh in the 1760s, Blagden displayed a fascination with the ‘association of ideas’, an interest also observed in the commonplace book he kept between 1769 and 1773. As this section will reveal, his approach to mind and memory may have informed the composition and use of his notebooks and diary.

The use of notes as *aides-mémoires* in the early modern period has been discussed by several historians. Ann Blair has connected note-taking with the concept of ‘information overload’, revealing how written notes supported memory in a variety of situations.^10^ In his work on Boyle's loose manuscript notes, Richard Yeo has pointed to their function in prompting recollection and relieving the memory, in addition to triggering trains of thought.^11^ Henderson has similarly suggested that Hooke's memoranda may have been prepared with the aim of jogging his memory.^12^ The use of notes and diaries as *aides-mémoires* was by no means confined to those with scientific interests. The late eighteenth-century landscape painter Joseph Farington kept a diary from 1793 to 1821, a period that overlaps with much of Blagden's own diary. When writing of his purpose in keeping a diary, Farington specifically noted its function as an aid to memory.^13^

Some early modern scholars turned to manuscript record-keeping to compensate for bad memory. Lotte Mulligan argues that Hooke, who was noted for his poor memory, compiled his memoranda to improve his ability to recall information, in line with his conception of memory as a store for impressions that could be improved by the act of compiling a daily record.^14^ By contrast, neither Blagden nor his contemporaries appear to have suggested that he possessed a bad memory. The biographer and diarist James Boswell remarked upon Blagden's ‘copiousness and precision’, suggesting that he possessed a good memory.^15^ Indeed Christa Jungnickel and Russell McCormmach argue that it was Blagden's ‘excellent memory for facts’ that made him appeal to those he worked for.^16^ Though historians have remarked upon Blagden's memory in such terms, no scholarship has yet explored the means by which he might have cultivated such abilities, or the models of memory that may have influenced his record-keeping.

Several of Blagden's notebooks reveal his interest in the association of ideas as a mechanism by which thoughts become connected in the mind, and may be prompted in sequence by a trigger. As Hugh Buckingham and Stanley Finger note, the notion of ‘triggering mnemonic movements in sequence’ is one that dates back to Aristotle's *De memoria et reminiscentia*, in which he distinguished between recognition memory, where previously encountered external stimuli cause one to recognize or remember previous experiences, and recollection, as the ability to search consciously through one's memory for something.^17^ Aristotle described recollection as a process whereby an individual deliberately hunts for something in the memory, seeking out traces ‘either similar, or contrary … or else … contiguous’ until the desired trace is found.^18^ In the eighteenth century, the philosopher David Hume, in *An enquiry concerning human understanding*, asserted that the mind contained ideas, images derived from sensory impressions, that could be connected to one another by ‘resemblance’, ‘contiguity in time or place’, and ‘cause or effect’.^19^ This notion was expanded upon by David Hartley, the philosopher and physician most often viewed as the forerunner to the school of association psychology.^20^

During his student days at Edinburgh, Blagden appears to have come into contact with Hume's conception of the association of ideas, as suggested by a small notebook dedicated to memory. In this notebook, Blagden wrote ‘M[emor]y. is faithful to the order of Ideas; hence train of think[in]g’, and recorded the ways in which ideas could be related to one another: ‘1. According to situation in time & place 2. resemblance in other qualities in wh[ich]. are diff[eren]t. degrees. 3 cause & Effect’.^21^ Though Blagden did not cite a source, these relations correspond exactly with those of Hume, namely ‘contiguity’, ‘resemblance’, and ‘cause or effect’. Hume was based in Edinburgh, and Blagden may well have met him during his time at the university.^22^

Evidence of Blagden's interest in the association of ideas is not confined to this particular notebook. On the very first page of his commonplace book, he mused that ‘Association of Ideas is the foundation of our ordinary train of thinking. If by any means the original idea is excited, the ideas connected by association present themselves in the order in which they were associated, to the mind.’^23^ Following this, Blagden reflected on an incident where he observed how a train of thought experienced during his youth was re-sparked by a recent visit to a church in Bristol, where he spent much time as a boy. He described how the sight of the church's chandeliers caused him to fall ‘into the same train of thoughts’ he had experienced while ‘listless at church’ as a child, where he daydreamed of shearing them from the ceiling using a magnetized sword.^24^ Though Hume did not refer to trains of thought in his description of the association of ideas, this statement suggests that, in Blagden's understanding, the concepts were closely linked when recalling events and thoughts from the past using a trigger, in this case a specific object in a familiar setting.

Though Blagden was much taken with the association of ideas, not all scholars of the seventeenth and eighteenth centuries considered it a productive mechanism for recollection. As Roger Lund argues, many saw associations between ideas based on similarity as potentially harmful to judgement and clear moral thinking.^25^ The philosopher Thomas Hobbes was concerned that those preoccupied with finding similitudes in the world, aligned with the possession of good ‘wit’, dissembled in making ‘things appear other than they are’.^26^ John Locke similarly feared that an ‘aptness to jumble things together wherein can be found any likeness’ could impair an individual's perception of the world.^27^ Hume acknowledged a similar concern, noting that, when ‘In our more serious thinking’ a connection to an idea is introduced from another that interrupts the ‘regular tract or chain of ideas’, it is ‘remarked and rejected’.^28^ Though Blagden commented upon ‘trains of thought’ in such terms, stating that where individuals failed to follow a ‘proper train’ this suggested ‘madness and delirium’ in opposition to rational thinking, his discussion of the association of ideas in recollecting previous events suggested the mechanism as a positive force for memory.^29^

Since his musings on associationism occur on the very first page of his commonplace book, one might suggest that Blagden was thinking specifically about memory and associations at the moment he began this manuscript. The diary that Blagden kept after his commonplace book may have operated as a prompt to recollection in line with his conception of the association of ideas, an assumption supported by evidence that Blagden re-read his diary when he sought to recall events. Some of the diary's entries feature annotations inserted after the original writing, one example being a note affixed to an entry dated 26 October 1794, ‘See [Wednesday] 29’. The later entry, which this note points to, provided further information on the events of 26 October, namely a conversation that Blagden had had with Lord George Macartney, recently returned from an embassy to China.^30^

Similarities between multiple diary entries and letters that Blagden composed around the same date further suggest the diary's use as a trigger for memory. One example can be found by comparing diary entries from his visit to Paris in 1802 with letters that he sent to Joseph Banks. In a letter dated 1 April, Blagden provided Banks with a detailed breakdown of all the papers read at a recent meeting of the Scientific Class of the Institut National. The diary was Blagden's repository for notes on each of these papers, as the entry for 27 March reveals:

there was first read an account of petrified palm wood found near Soissons in sand under limestone of [*sic*] shells in it. Some remarks by the author not well founded but the petrified stone was really palm wood, then a memoire by Seguin on Hungary leather, that made with alum.^31^

Viewed alongside passages concerning the Institut in the later letter to Banks, a clear resemblance is seen:

At the meeting of last Saturday the first paper read was on the subject of fossil petrified palm wood found near Soissons; it was really palm wood, & had the genuine structure of that tribe of vegetables very evidently, tho’ the whole was stone, I believe impure siliceous stone; but we have seen the like from a great many places. This was found in sand, under limestone. [*illegible word*] memoires then read by Mr Seguin, on the method of tanning with alum.^32^

The detail in the letter elaborates slightly on that found in the diary, but in places includes identical wording, such as ‘was really palm wood’. The fact that Blagden mentioned the structure of the palm wood, though no such note featured in the diary, suggests that the diary may have succeeded in prompting recollection.

In his commonplace book, Blagden noted the power of objects in re-prompting trains of thought, but the same mechanism might have applied when reading words on a page. One might consider Hume's discussion of associative links forged by ‘contiguity’, observed in the way that ‘the mention of one apartment in a building naturally introduces an enquiry or discourse concerning the others’, suggesting the power of recording the first item in a sequence in leading one to recall the items that follow.^33^ In stating only the beginning or topic of an idea or conversation, Blagden's diary may have been calculated to contain all that was needed to prompt previous trains of thought, suggesting one explanation for its terse prose.

## Case notes and commonplace books: Blagden's medical note-taking

Blagden began his diary in the 1770s, but had experimented with various genres of record-keeping prior to this. This section briefly examines three such genres—lecture notes, case notes and a commonplace book—produced in aid of his efforts to pursue a medical career in the 1760s and 1770s. Born into a Gloucestershire mercantile family, Blagden possessed a meagre fortune, describing his father as having ‘lived profusely’ and thus left little to the family.^34^ In becoming a physician, he hoped to secure greater personal fortune, alongside social status, by moving from Gloucester to a more ‘eligible situation’ in London, where he expected a physician might earn between £2000 and £4000 a year.^35^ In the rest of this paper, I will show how several manuscript genres connected with Blagden's career and ambitions contributed to shaping the diary.

After graduating MD from Edinburgh University in 1768, Blagden attained a practice in Gloucester.^36^ Several years later, he obtained a position as an army surgeon, and in 1776 sailed for North America to serve on board a hospital ship during the American War of Independence, a move he hoped would raise his reputation and provide ‘the most effectual & honourable introduction to Practice in London’.^37^ A physician's training involved the compilation of vast quantities of handwritten notes from lectures and reading, providing Blagden with a high level of training in observation and recording. Matthew Eddy, who has explored Blagden's medical note-taking, argues that many students were ‘shaped profoundly’ by their experiences, their courses establishing ‘scribal abilities and values’ that endured throughout life.^38^ Blagden's student manuscripts instilled in him a discipline for daily writing, a habit that continued until his death in 1820. While Blagden began his diary some years after graduating, two of his Edinburgh contemporaries, the physicians Sylas Neville and Benjamin Rush, kept diaries as students, suggesting a plausible link between the need to make notes as part of medical training and the desire to keep a diary.^39^

In addition to lecture and reading notes, medical training also involved the production of case notes, a genre of writing with clear similarities to the diary. Case notes and histories were compiled by students from observation of patients, which, as Julia Epstein notes, became a key part of a physician's training in the eighteenth century.^40^ As Epstein argues, case notes possess a narrative as ‘a chronology of bodily events’, reported by the patient and recorded by the physician.^41^ In Blagden's case notes, made from the patients he saw at Edinburgh hospital, entries began with the patient's admission date and details at the top of the page, and proceeded chronologically, recording changes in symptoms and prescribed remedies as they occurred.^42^

**Figure 1.**
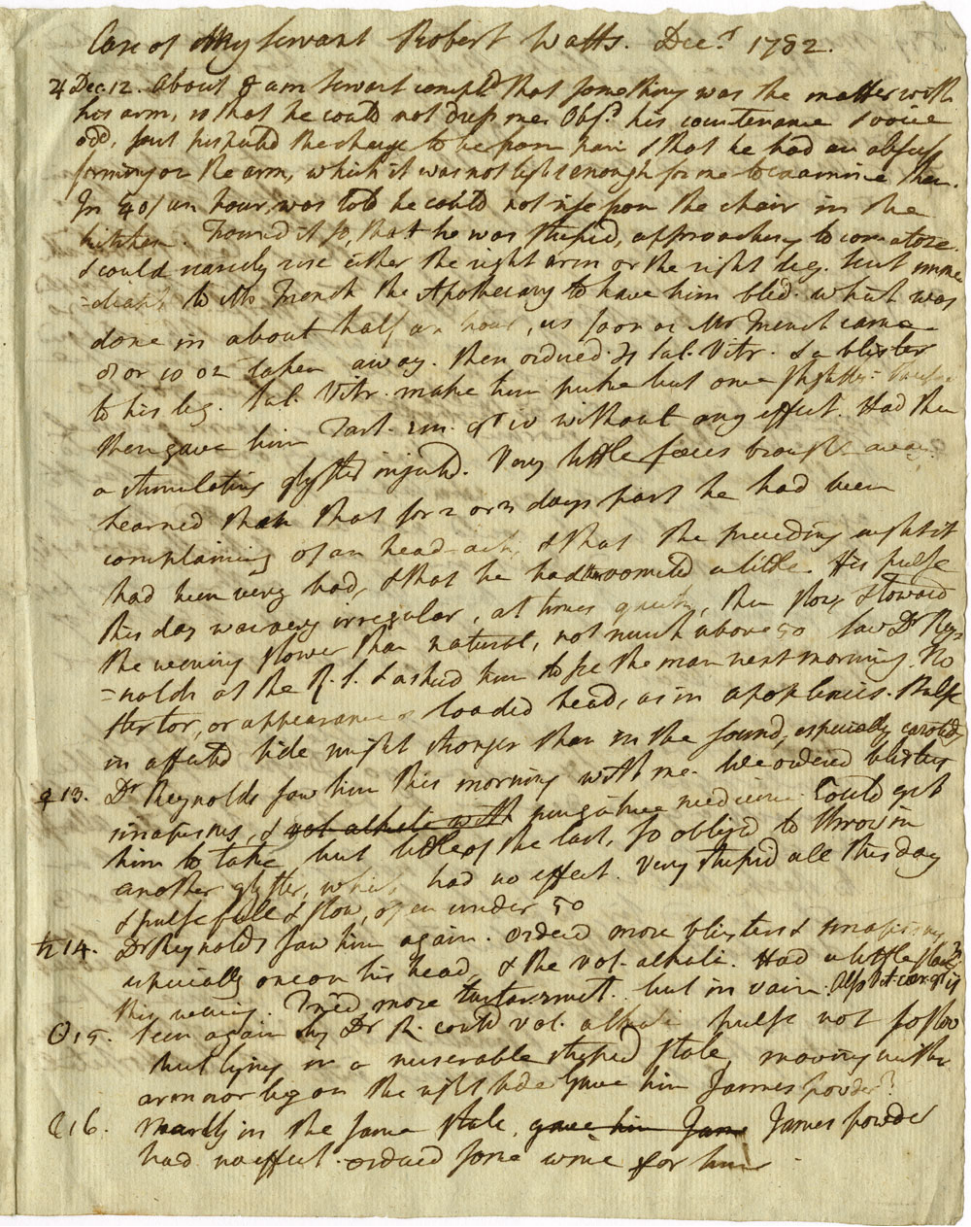
Case summary written by Blagden on the illness of his servant Robert Watts, dated December 1782 (Royal Society, CB/4/5, BLA.5.2). Copyright © The Royal Society.

**Figure 2.**
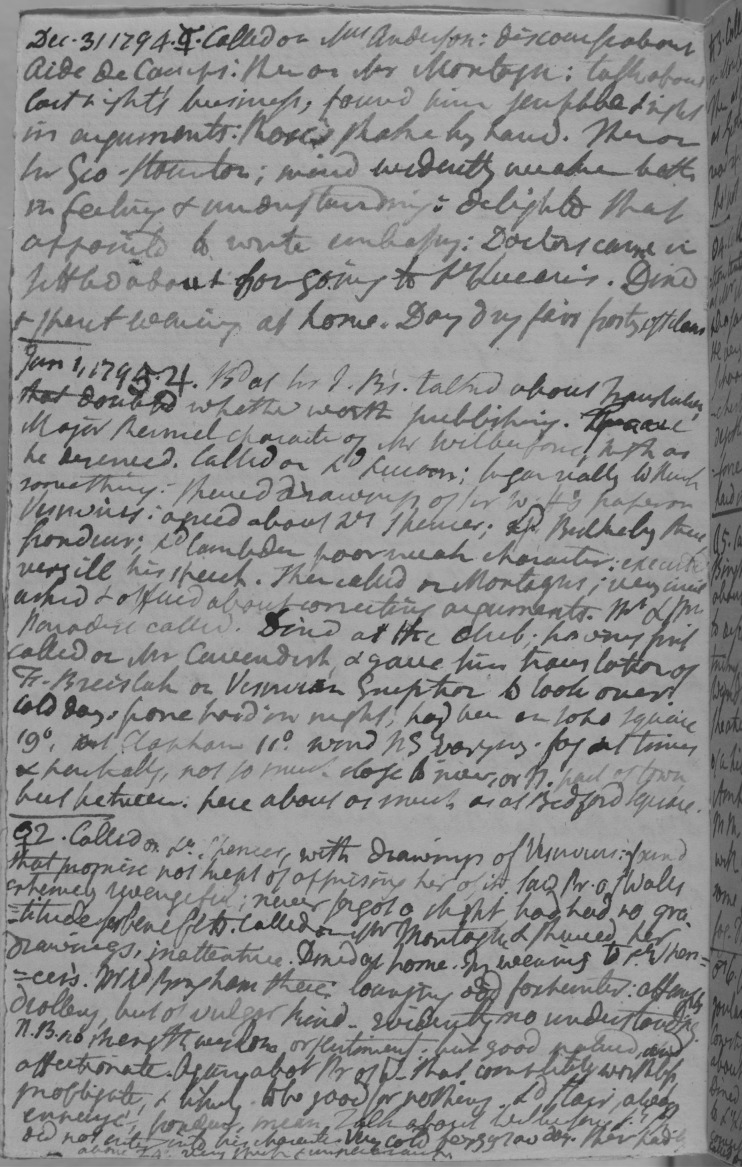
Diary entries written by Blagden for 1 and 2 January 1795 (Royal Society, CB/3/3, f. 40v). Copyright © The Royal Society.

In addition to case notes, Blagden compiled longer summaries of particular cases of interest, which bore close resemblance to the form of his later diary. Some of these summaries date from immediately before his service as surgeon to the army, suggesting that he may have produced them as part of his professional medical work.^43^ However, he continued to compile such summaries after he ceased practice as a physician in the 1780s.^44^ Within the Royal Society's collection of Blagden's medical notes is a large sheet headed ‘Case of My Servant Robert Watts. Dec. 1782’ (Figure 1), which displays structural similarities to Blagden's later diary (Figure 2).^45^ The summary breaks with the form of Blagden's earlier student case notes, suggesting that, after his graduation, his medical notes were adapted to suit his needs, and were in turn influenced by the form of his diary. The case of Robert Watts features the date down the left-hand side of the page, accompanied by the astrological symbol for the day of the week, a convention that Blagden began using in his diary in 1776, but that he had not used in his Edinburgh student notes. Rather than providing brief notes of symptoms and remedies as in the style of his student notes, he gave a fuller prose account of Watts's illness, along with actions taken each day, closely resembling the form of his diary.

After graduating, and during his time as a physician in Gloucester, Blagden kept a commonplace book, a genre of manuscript traditionally used for organizing notes, typically from personal reading, with the aim of collating useful knowledge for future deployment in writing and speech. As David Allan argues, ‘commonplacing’ can be traced back to antiquity, and to Aristotle and Cicero's extraction of arguments to be used and applied to multiple cases.^46^ During the Renaissance, the genre gained pedagogical associations, with note-takers advised to identify themes in their reading and to structure their notes accordingly, thus aiding their recovery and re-use.^47^ Ann Moss argues that such organization was intended to encourage recollection of related discourse from various authors.^48^

Though some historians have suggested that the eighteenth century saw the decline of the commonplace book, Allan argues that the tradition continued, and that the genre became more flexible and diverse. He notes that some commonplace books were seen by their owners as ‘little more than convenient *aides-mémoires*’, used to store information from personal experience, in addition to notes from reading.^49^ Blagden used his own commonplace book to record reading notes, personal musings, visits to friends, conversations and patient histories.

Blagden's commonplace book did not feature a thematic index, as associated with earlier incarnations of the genre. Instead, he often entered his notes alongside the date, a convention he continued in the form of his diary.^50^ His student contemporary Benjamin Rush, who also compiled a commonplace book, followed the same practice in entering notes alongside the date.^51^ Such temporal indexing was not anathema to methods of note-taking in the eighteenth century. As Yeo observes of a number of Locke's manuscripts (notably his ‘Adversaria physica’ and one of his commonplace books), if the source of the material he sought to record was not a book, Locke noted the information in a dated entry.^52^ Some note-takers explicitly compared their practices to those of mercantile record-keeping, and in particular the genre of the ‘waste book’, in which merchants recorded the day's transactions in order of occurrence. Blair notes Francis Bacon's categorization of his notebook as akin to a ‘merchant's waste book, where to enter all manner of remembrance of matter, form, business, study, touching my-self, service, others’.^53^ Note-taking conducted in this manner possessed similarities with the form of a diary, as a diurnal record.

Despite its lack of a thematic index, Blagden's commonplace book arguably owed a key feature of its use to the tradition of commonplacing in preserving information for later application. The recording of anecdotes and information of value is observed in the manuscripts of another eighteenth-century scholar, the antiquary Thomas Hearne, who recorded notes from reading, descriptions of antiquities, drafts of letters, and daily events in his diary.^54^ The recording of information in this manner could be useful for a variety of endeavours; Elizabeth Yale has noted the strong affinity between Baconian ‘incremental fact gathering’, associated with both natural history and antiquarianism, and the use of manuscripts to collect, organize and share information.^55^ Hearne's diary features comments such as ‘July 10 [1705] … One of the Graecians of Glocester [*sic*] Hall tells me w[he]n he came out of his Country he brought with him a Greek M[anuscrip]t written above a thousand years since’.^56^ This comment is similar to notes made in Blagden's commonplace book that document curious facts worthy of investigation, such as ‘One James, in Shoe Lane, has a curious collection of Pidgeons [*sic*] which it would be worth while to see’.^57^

In its dated entries and autobiographical detail, Blagden's commonplace book appears to be his first experimentation with the diary form. As the relative flexibility of the form of the eighteenth-century commonplace book might suggest, conceptual boundaries between diaries and commonplace books were arguably slightly permeable. Yet, rather than choosing to continue his commonplace book, Blagden began a diary. Though the diary possesses some similarities with the commonplace book, in its inclusion of useful anecdotes and conversations indexed by date, some aspects of the commonplace book's content feature far less frequently and often not at all, including notes from reading and case histories. The two manuscripts also differ in their appearance. While the commonplace book is a hefty leather-bound volume, the diary consists of small folded sheets, infinitely more portable than a large bound book. As I will suggest below, the form and content of Blagden's subsequent diary was influenced by his decision to pursue a different career in the mid 1770s.

## Blagden as traveller and collector: voyage narratives and patronage in the early diary

In 1776, Blagden began keeping a regular diary, coinciding with his service as surgeon to the army between 1776 and 1780 on board the hospital ship *Pigot*. At this time, he also began to cultivate the patronage of Joseph Banks and the Royal Society, having been elected Fellow in 1772. Banks was a landed gentleman who rose to international celebrity and scientific prominence in the wake of his participation in Captain James Cook's *Endeavour* voyage from 1768 to 1771.^58^ During Blagden's career as a surgeon in America, Banks's celebrity made him an obvious choice of patron for someone seeking to raise their standing in the social and scientific worlds. As Banks noted several years after his association with Blagden had begun, Blagden expressed ‘humiliation’ at claiming ‘professional reward’ and income from his work as a physician.^59^ Through his connection to Banks, Blagden arguably aimed to pursue a career more conducive to the status of a gentleman, perhaps in imitation of Banks's own career, rather than that of a medical ‘professional’ employed by patients.^60^

Between 1776 and 1780, contemporary interest in voyage and travel narratives, fuelled by the return of celebrated voyages such as Cook's, provides one possible context for the development of Blagden's diary, as a form suited to the collation of observations used to win patrons and to bolster one's image as a gentleman of science. This section will reveal how Blagden's desire to pursue gentlemanly status through Banks's patronage, achieved by sending gifts of information and specimens, was supported by the developing form and content of his diary.

Prior to and during the composition of his diary, Blagden was an avid reader of travel literature. Evidence of his reading can be found in a collection of papers entitled ‘Travel records and observations’, held within the Beinecke Library. Among these papers are several sheets containing notes extracted from a variety of travel texts, including Thomas Pennant's *Tour in Scotland 1769* (1771), William Borlase's *Natural history of Cornwall* (1758) and Andrew Burnaby's *Travels through the middle settlements in North America* (1775).^61^

Blagden's reading notes reveal a fascination with topics that in turn feature heavily in his own writing, including landscape, industry and local customs. In his reading of Pennant, he extracted notes relating to landscape and local activities, often blurring the two in a manner mirrored in his own recording of his time spent travelling. When reading the *Tour in Scotland*, he extracted a quotation from Pennant's description of a particular journey, ‘From Glen Tilt. Ascend a Steep hill, & find ourselves on an Arrie, or tract of mountain which the families of one or two hamlets retire to with their flocks for pasture in summer.’^62^ The earliest portion of Blagden's diary contains similar descriptions, where local activities are described alongside the landscape. On 23 January 1776, he wrote: ‘Saw today a cave running in toward the bottom of this bay the separation about ¼ of a mile: called barly cave.’^63^ In the same entry, he made a record of local activities in Crookhaven, the coastal town on the southern-most tip of Ireland where the *Pigot* moored before the journey to America: ‘Mr Malony's public house at crookhaven a most notorious bawdy house 5 girls of all prices.’^64^

While similarities can be traced between the content of travel narratives and Blagden's diary, the structure of the diary's entries also reveals the influence of contemporary travel writing on his methods for storing information. In several aspects of its form, the diary resembles the structure of eighteenth-century voyage narratives that sought to replicate the appearance of a ship's log, compiled to record position, direction, speed and weather.^65^ Blagden's diary for the years he spent on board the *Pigot* contains several features reminiscent of a ship's log. Entries frequently contained notes on temperature, weather conditions and astronomical observations, as in that for 19 February 1777:

At 8 ½ a. m. Ther[mometer]. 18 Bar[omete]r 29.77. Wind northerly, blows very sharp … By altitudes reflected horizon found that at ½ after 9 in morning watch, which had been just wound up, was 0h 35′ 11″ too slow for apparent time … Then observed Transit of Bear's Tail at 5.h 39.′ 8″ by Watch.^66^

Though unsurprising for a diary kept while at sea, it is striking that notes on weather and temperature feature throughout Blagden's diary. Entries made during the 1790s, when he spent the majority of his time in London, invariably begin or end with a mention of the day's weather, as seen in the entry at the beginning of this paper.

While weather recording in Blagden's early diary may have been influenced by travel literature and his experience of voyages, such practice is also found in the manuscripts of earlier natural philosophical diarists and note-takers. Hooke's memoranda, which took the form of daily entries, began as a record of the weather alongside personal observations.^67^ Hooke, Boyle and Locke all recorded weather observations in connection with their interests in the effects of climate on bodily health.^68^ As Jan Golinski notes, connections had been drawn since antiquity between weather and well-being, a practice that continued with many eighteenth-century weather diarists recording the prevalence of disease alongside temperature, moisture and pressure at specific locations.^69^ Blagden's diary reveals a related interest, concerning the links between landscape and health. During a trip to south-west England in the summer of 1780, he recorded impressions of the health of the residents of Glastonbury in relation to its soil, noting it as ‘almost surrounded with clay, & people there look by no means healthy … I was told of many agues’.^70^

Alongside details concerning time and temperature, Blagden described local flora and fauna in his diary, topics also found within the notes he extracted from travel narratives.^71^ The diary composed during his service in North America features extensive descriptions, including measurements made while examining creatures. On 23 January 1776, he recorded the following:

Grey plovers very plenty on banks of crook haven: measure 10 inches from vertex of head to extr[emit]y of tail 1 foot 9 inches from tip of wing to tip: 8 inches round the chest part of body: 2 inches from tip of beak to center of head beak 1 inch from tip of middle toe to joint of thigh 6 inches: back & back of head & neck brown speckled variegated with ferruginous white.^72^

The form of the diary during the 1770s, shaped by Blagden's situation as a traveller and his familiarity with travel writing, assisted his efforts in embarking upon a patronage relationship by cultivating the interest of Joseph Banks. Though Blagden and Banks had corresponded prior to Blagden's departure for North America, analysis of their letter exchange reveals that during his military service their correspondence intensified, concurrently with the inception of the diary.^73^ This correspondence contained news of the war and Blagden's progress in collecting for Banks, a task for which Blagden routinely stored information in his diary.^74^

The role of gifting items to established collectors in exchange for social distinction has been explored by scholars in relation to the networks of the early eighteenth-century president of the Royal Society Sir Hans Sloane. James Delbourgo notes that Sloane's social prestige rendered him highly attractive to individuals in search of reward and advancement.^75^ Gifts to Sloane were often unsolicited, and were offered by those seeking to ‘collect the collector’ as a patron, an art form requiring skill and discernment in the selection of suitable gifts that might result in the elevation of an individual's standing through Sloane's patronage.^76^ In sending specimens and news to Banks, Blagden hoped to garner favour in a similar manner, with the aim of capitalizing on his efforts upon his return to Britain, when he immediately, though unsuccessfully, requested that Banks secure a position and lodgings for him at the Royal Society.^77^

The diary enabled Blagden to document information and objects useful in winning Banks's favour. One gift that Blagden sent was a collection of more than 100 specimens of mammals, birds and fish, collected in Rhode Island.^78^ This collection had originally been commissioned by the antiquary Daines Barrington on behalf of his friend the collector Ashton Lever. However, Blagden saw the opportunity as one in which he might appeal to Banks, his preferred patron, by choosing to send the collection jointly to both Barrington and Banks, and by flattering Banks: ‘Mr Lever wants any thing that he happens not to have in his Museum … on the contrary nothing can be an object to you [Banks] but what will conduce to the improvement of Natural History.’^79^

Blagden recorded this collection in his diary, noting the specimens he observed and caught on particular days, alongside measurements, behaviour and preparation methods. While the recording of information concerning local fauna was something that he had encountered in his reading of travel narratives, his precision recording of specimens would here have assisted him in collating data before sending it to Banks. Information concerning these specimens also featured in a manuscript catalogue in condensed form, indexed by date of capture. The diary and catalogue may then have been consulted, either singly or together, when Blagden wrote to Banks in September 1778 to provide him with a list of the specimens.

An example of how this may have operated in practice can be given with reference to a particular specimen. A bird identified as a species of ‘diver’ was collected by Blagden on 18 February 1777. In his diary entry for that day, he recorded, ‘a fine Colymbus, which I put into spirits with a piece of sack-thread tied to one foot, with 2 knots in it. the bird was swimming with three others near the beach: belly very thick of feathers, & a most glossy, silvery white’.^80^ In the catalogue, this creature was recorded as ‘2. Colymbus. Small White Belly. Shot by Mr Scott. Feb[ruar]y 18’.^81^ In the letter that Blagden sent to Banks on 12 September 1778, the same specimen can again be identified: ‘No 2 (A Bird) A species of Diver; the breast of a most beautiful glossy white when it was shot: swam in company with two others: no particular name here but Loon. Feb[ruary] 18’.^82^ This bird is clearly the same across all three manuscripts, its identification as specimen number two confirmed on the body of the bird by the two knots in the sack-thread attached to its foot. One difference, almost certainly a transcription error, is the number of birds described as swimming in company with this individual; while in the letter Blagden referred to two, in the diary he mentioned three. The fact that he recalled the local name of the bird as ‘Loon’ in his letter to Banks, though no such note was made in either the catalogue or the diary, suggests that his brief notes may have prompted the recollection of further details.

## Blagden as secretary and networker: managing employment at the Royal Society

In 1784, Blagden was elected secretary of the Royal Society, a position that resulted in no small part from his efforts to become Banks's client.^83^ While he served as secretary, the diary assisted in his efforts to manage his employment. In this context, it was the activities of the Society and its Fellows, rather than local wildlife, that featured most heavily in the diary. At this time, the diary continued to serve Blagden's aspirations for social advancement through proximity to the Society and Banks, by functioning as a record of his engagements and various secretarial duties.

The diary that Blagden kept during this period took the form of an itemized record of events, places and people. It was used to keep track of daily affairs, social interactions, financial transactions and his itinerary. A portion of an entry dated 3 March 1795 illustrates this process:

[Tuesday] 3. Called on Montagus. Mrs M[ontagu]. character of Leckie: vile without sentiment most odious: then on Lord Lucan, very civil: Del Campo there asked tomorrow … Left at Banker Bill for £1800 on Witcheads. Letter from T[homas] B[lagden] with that bill inclosed.^84^

In this entry, Blagden recorded visits to two of his acquaintances, Matthew Montagu, MP and adopted nephew of the celebrated bluestocking Elizabeth Montagu, and Sir Charles Bingham, first Earl of Lucan. He noted the rough topics of conversation during his social engagements, including the disposition of an unknown individual, ‘Leckie’, in addition to the other attendees at the houses of his friends, including the Marquis del Campo, a Spanish ambassador favoured by Queen Charlotte.^85^ A financial transaction is also mentioned, a deposit of £1800 with Blagden's banker, a sum sent to him by his brother Thomas, for a legacy received from the death of a family member, most probably his uncle.^86^

In its itinerary-style composition, the diary of Joseph Farington presents an interesting comparison with Blagden's diary. Farington was an active member of the Royal Academy and a Fellow of the Royal Society of Antiquaries, alongside his work as a topographical artist.^87^ Regarding his purpose in keeping a diary, Farington noted that the manuscript was intended ‘to assist my recollection in matters in which I was engaged’, suggesting its function as a tool for managing activities and employment.^88^ His entry for 31 July 1793 reveals a similar approach to writing to Blagden's, in its itemization of activities, people and weather:

July 31st. Rose at 7. A wet morning. Outlining view of High Street, Oxford, on Canvass [*sic*]. Called at the Shakespeare Gallery. –News of the taking Valenciennes. Dined at the Bedford Coffee House, with Messrs Berwick, D & S. Lysons, and H. Hammond. Excellent Port wine –Called in the evening with Berwick & S Lysons on G: Dance, to see the profile Heads. –Fine day. Glass rose to 71.^89^

Such use of a diary is not dissimilar to Blagden's, and suggests that this format was useful for those engaged in the lifestyle of a gentleman about town.

Serving as a record of the day's occurrences, Blagden's diary may have provided assistance in managing the tasks of secretary of the Royal Society. The role of the secretary in the Society's first statutes was codified as including ‘care of [the Society's] books, documents and correspondence’, attendance at all meetings of the Society and council, the production of notes and minutes pertaining to meetings, and the drawing up of all letters written on behalf of the Society.^90^ After the institution of the Society's journal, the *Philosophical Transactions*, in 1665, the preparation of papers for publication was added to the secretary's duties.

In several entries, Blagden recorded key aspects of the Society's business. Entries frequently referred to corrections and discussions of submitted papers, and to the management of correspondence with authors and Fellows.^91^ The diary also assisted Blagden in keeping track of those with whom he had shared drafts of papers outside the Society's meetings, in times when he needed a second opinion. He frequently sought such advice from his friend Henry Cavendish, as recorded in an entry for 19 October 1795: ‘Went to Mr Cavendish, discussed paper on light; found [I] had read it imperfectly; he advised recommending author to render [the paper] more compact & correct.’^92^

The diary served as Blagden's personal record of the activities and attendance of the Society's Fellows, in the brief notes he made of meetings of the Society and its dining club at the Crown and Anchor public house. Entries frequently mentioned notable attendees, alongside their behaviour, as in one example taken from 19 November 1795: ‘Dined at Crown & Anchor. L[or]d Palmerston; was rather civiler. Sir W[illiam]. Young's curious papers about West Indies.’^93^ Though such notes were not exhaustive in their description of attendees and conversations, they do provide additional insight into the activities of the Society, and in particular that of its dining club. Unlike the ordinary meetings of the Royal Society, recorded in the journal book, only the names of members and their guests were recorded from the meetings of the dining club.^94^ In this respect, Blagden's diary gives insight into Society activities otherwise unrecorded.

## The ‘Elevated ranks of Society’: female patronage and gentlemanly status

Though in his early career Blagden had been keen to foster Banks's patronage as a means of securing social and scientific advancement, it was during his time as secretary that he suffered a breakdown in his patronage relationship, centred on dissatisfaction at the way in which the relationship operated. In the late 1780s, Blagden experienced a rupture with Banks, tied to concerns surrounding the menial and ungentlemanly nature of his position as secretary, and in 1797 he resigned from the role. The diary reveals that, after this, Blagden attempted to cultivate a patronage network beyond Banks and the Royal Society, composed of a number of aristocratic women. In this endeavour, the form and content of the diary, developed in relation to his medical training, service in North America and work as Royal Society secretary, was suited to the recording of information when pursuing a new network of patrons. Though Blagden's attempts to build a patronage network cannot have been solely dependent on his practices of record-keeping, the diary may nonetheless have facilitated exchanges aimed at fostering a network of well-heeled patrons.

During his dispute with Banks, Blagden revealed his unease at his lowly status as a salaried assistant and secretary. The position of secretary was one that provided the post-holder with financial reward, in the form of an honorarium.^95^ As Steven Shapin has discussed in relation to the role of the ‘invisible technician’, payment in a working relationship entailed the subordination of the employee to the employer, in an exchange of authority and autonomy for financial reward.^96^ The initial indications of a break in Blagden's relationship with Banks occurred in letters sent in early 1788, in which Blagden wrote of his desire to resign as secretary. On 2 February, he said that he wished to continue promoting ‘the pursuits & interests of the Royal Society in the same manner’ but that, from now on, he intended to conduct this work as a ‘voluntary act’, rather than as a paid employee.^97^ His desire for the status of a gentleman, possessing fortune and social standing, was arguably incompatible with work as a salaried assistant. As the dispute continued, Blagden revealed his discontent at a lack of reward. Writing in April 1790, he complained that he had not received the benefits he expected as Banks's client, ‘namely the improvement of one's fortune, and the advancement of one's situation in society’.^98^

Though Blagden continued as secretary until 1797, tensions with Banks persisted. It is clear from the diary that, during the 1790s, he had begun to seek patronage elsewhere. Immediately prior to Blagden's resignation, Banks implied that some Fellows had become troubled by his ‘absences’ from duty, occasioned by his ‘habits … lately adopted of mixing much in the Gay Circles of the more Elevated ranks of Society’.^99^ Blagden's diary from the late 1780s and early 1790s reveals that he had begun to engage a network of individuals within these ‘Elevated ranks’, including the Duchess of Devonshire, Georgiana Cavendish; Lady Lavinia Spencer, the wife of the 2nd Earl Spencer; the bluestocking Elizabeth Montagu; and Lady Margaret Lucan, the wife of Charles Bingham, 1st Earl of Lucan and mother to Lavinia Spencer. Blagden corresponded with further aristocratic women, including Lady Elizabeth Grey, wife of the 1st Earl Grey, and Lady Mary Palmerston, second wife of Henry Temple, 2nd Viscount Palmerston.^100^

Interactions with these women involved the exchange of information and company for benefits which Blagden found more conducive to his social standing than those derived from his association with Banks. As correspondents and acquaintances, these women received scientific news. In correspondence with Lady Grey, Blagden summarized the latest scientific papers published in the *Philosophical Transactions*, at Lady Grey's request.^101^ On other occasions, these women requested Blagden's company and expertise; in November 1793, Georgiana Cavendish asked that he accompany her on a tour of the British Museum.^102^ Blagden also attended these women at their own homes, at elegant gatherings or ‘assemblies’, where news and information pertaining to a variety of fields, including natural philosophy, were shared and exchanged in the company of invited men and women.^103^ At times, he was called upon to give medical advice, his expertise continuing to be valued long after he ceased practice as a physician. On 24 June 1795, he recorded a visit he made to Elizabeth Montagu to examine her servant, recently injured by a fall.^104^ Having first met Blagden at a party at Portman Square in 1788, the author Hannah More described him as ‘a new bluestocking, and a very agreeable one … Willing to teach, and yet not proud to know’.^105^ More's comment exemplifies Blagden's role among these women as a convivial provider of interesting news and ideas.

Though Blagden does not appear to have benefited financially from this network, such interactions bolstered his standing as a gentleman. In attending these female-run assemblies, he made acquaintances among London society, all the while enacting the role of the clubbable gentleman about town, in addition to gathering news. On 3 April 1795, he recorded in his diary a typical visit to Lady Lucan's, during which he met notable members of the aristocracy, Lord Palmerston and Lord Lucan, ‘In even[in]g. to L[ad]y Lucan's … L[or]d Palmerston came in. L[or]d L[ucan]. mentioned that he was going with L[ad]y Camden to Ireland.’^106^ In his work with and for Banks, Blagden had also encountered members of the aristocracy on a regular basis, as a frequent attendee of Banks's breakfasts at his Soho Square residence, and as his occasional advisor at meetings of the Privy Council.^107^ What was different about Blagden's relationships with these aristocratic women was that they were not formalized in the same manner as his association with Banks. As a client and acquaintance of elegant ladies, Blagden was neither paid nor operating under the title of secretary, rendering his services voluntary actions rather than formal duties, supporting his image as a gentleman.

In all these engagements, Blagden's diary may have facilitated his remembrance of news and meetings, and the information and objects suitable for presentation to multiple patrons. The entries tracked flows of the same information to various individuals, as in one example concerning the drawings for William Hamilton's paper on the eruption of Vesuvius, published in the *Philosophical Transactions* in 1795. On 1 January, Blagden recorded ‘Called on L[or]d Lucan … shewed drawings of Sir W[illiam]. H[amilton]'s paper’, before mentioning the same illustrations on 2 January: ‘Called on L[ad]y. Spencer, with drawings of Vesuvius … Called on Mrs Montagu & shewed her drawings, inattentive.’^108^ As this extract reveals, at times he was unsuccessful in cultivating the interest of his patrons using gifts of knowledge and objects. As Blagden made the transition from being the client of a single male patron to one engaged by a network of individuals beyond the Royal Society, these interactions shaped the form and content of the diary. Moreover, his training in the use of the diary as a repository for gift-worthy information, and as a record for managing its flows, suggests the diary's potential utility in fostering his reputation as a learned gentleman who provided news, objects and entertainment to a network of aristocratic patrons.

## Conclusion

Blagden's diary operated as a tool for the management of information, and developed in its form, content and structure alongside his career and social ambitions. His record-keeping practices were arguably influenced by an interest in memory and recollection, centred on the association of ideas. The diary compounded several manuscript genres that Blagden encountered during his early life as a student and physician. His familiarity with further forms of diurnal recording, in his commonplace book and in travel writing, further shaped the form of the diary in content and organization, which in turn assisted him in pursuing solutions to his social ambitions. When following systems of patronage, his diary was of use in recording the types of information that could be gifted to patrons, in addition to managing his work as secretary of the Royal Society. This examination of Blagden's diary has revealed one solution to the management of information and social ambition, shaped by patronage.

Henderson notes that Hooke undertook his diary writing in order to ‘record things that he felt needed to be remembered’, including ‘work, money, books, natural philosophy, news, health and household affairs’.^109^ Blagden's diary, composed a century later, fulfilled a similar function, in operating as an aid to memory concerning all facets of his personal life and career. In its nature as a material manifestation of Blagden's interests and ambitions, the diary appears similar to Elaine Leong's assessment of early modern recipe books. Leong argues that these served as ‘testaments of the interests and needs of particular families’, shaped by the social and cultural contexts surrounding their composition.^110^

As this paper has suggested, Blagden's diary was shaped by transitions in patronage aimed at providing a solution to social ambitions. At the same time, it assisted him in pursuing particular relationships and careers, in its role as an *aide-mémoire*. Blagden turned to a dispersed patronage network, rather than that of a single male patron, in his preoccupation with achieving the status of a gentleman. While recent work has exposed the networks of patronage over which male scholars such as Joseph Banks and Hans Sloane presided, little has been explored regarding the networks of aristocratic women engaged in scientific learning and patronage.^111^ As a study of the diary has revealed, female patronage could trump that offered by men, in so far as the setting of female ‘assemblies’ offered a situation in which Blagden was not cast as the inferior assistant of a male scholar in a formal institution, but as an independent gentleman contributing to discussions headed by female patrons.

This paper has extended the current historiography that considers early modern solutions for information management, by considering the development of a manuscript over time, and in relation to social ambition and career advancement, assisted by the form of a personal diary. As such, Blagden's diary might be said to constitute a ‘sociomaterial’ object, in its nature as a manuscript tied to its author, wherein the identities of both object and person were co-constituted over time.^112^ As Blagden developed the diary in form and content, so the diary interacted with his persona, career and relationships, all in the service of personal advancement. Though this paper has sought to explore the diary in its own right, it has revealed that Blagden and the diary were inextricably linked.

## Footnotes

1 Royal Society, CB/3/3, f. 40v (Charles Blagden, diary entry, 1 Jan. 1795).

2 Clara Anderson, ‘Blagden papers at the Royal Society’, *Notes Rec. R. Soc.* **61**, 237–238 (2007).

3 See Royal Society, MS/821 (additional papers of Charles Blagden).

4 See Beinecke Library, Yale University, Osborn fc16 (Sir Charles Blagden, diaries, 1776–1788) and OSB MSS 51 (Sir Charles Blagden papers).

5 Danielle Fauque, ‘An Englishman abroad: Charles Blagden's visit to Paris in 1783’, *Notes Rec. R. Soc.* **62**, 373–390 (2008).

6 Iain P. Watts, ‘Philosophical intelligence: letters, print, and experiment during Napoleon's Continental Blockade’, *Isis* **106**, 749–770 (2015).

7 For Blagden's uses of the term ‘information’, see Royal Society, CB/3/3, ff. 45v, 57r, 69r (Charles Blagden, diary entries, 15 Jan., 4 May and 7 Oct. 1795).

8 Michael Hunter and Charles Littleton, ‘The work-diaries of Robert Boyle: a newly discovered source and its internet publication’, *Notes Rec. R. Soc.* **55**, 373–390 (2001).

9 Felicity Henderson, ‘Unpublished material from the memorandum book of Robert Hooke, Guildhall Library MS 1758’, *Notes Rec. R. Soc.* **61**, 129–175 (2007), at 131.

10 Ann Blair, *Too much to know: managing scholarly information before the modern age* (Yale University Press, New Haven and London, 2010).

11 Richard Yeo, *Notebooks, English virtuosi, and early modern science* (University of Chicago Press, 2014), pp. 152–173.

12 Henderson, *op. cit.* (note 9), p. 132.

13 Joseph Farington, *The diary of Joseph Farington* (ed. Kenneth Garlick and Angus Macintyre) (Yale University Press, New Haven and London, 1978), vol. 1, p. xxxiii.

14 Lotte Mulligan, ‘Robert Hooke's “Memoranda”: memory and natural history’, *Ann. Sci.* **49**, 47–61 (1992).

15 James Boswell, quoted in Christa Jungnickel and Russell McCormmach, *Cavendish: The Experimental Life*, revised edn (Bucknell, Lewisburg, PA, 1999), p. 294.

16 Jungnickel and McCormmach, *op. cit.* (note 15), p. 294.

17 Hugh W. Buckingham and Stanley Finger, ‘David Hartley's psychobiological associationism and the legacy of Aristotle’, *J. Hist. Neurosci.* **6**, 21–37 (1997), at 23.

18 Aristotle, quoted in *ibid*., pp. 23–24.

19 David Hume, *An enquiry concerning human understanding, reprinted from the edition of 1777* (Open Court Publishing Co., Chicago, 1900), pp. 21–22. Buckingham and Finger, *op. cit.* (note 17), p. 26.

20 Buckingham and Finger, *op. cit.* (note 17), pp. 26–35.

21 Wellcome Library, London, MS/1250 (Charles Blagden, notebook on memory, undated).

22 The diary of the politician and physician Benjamin Rush, a contemporary of Blagden's at Edinburgh, reveals that Rush met Hume at a dinner hosted by the Scottish physician Sir Alexander Dick. See Benjamin Rush, *The autobiography of Benjamin Rush: his ‘Travels through life’ together with his commonplace book for 1789–1813* (ed. George W. Corner) (American Philosophical Society, Philadelphia, 1948), p. 49.

23 Wellcome Library, MS/1245 (Charles Blagden, commonplace book, undated entry).

24 *Ibid*.

25 Roger D. Lund, ‘Wit, judgement, and the misprisions of similitude’, *J. Hist. Ideas* **65**, 53–74 (2004).

26 Thomas Hobbes, quoted in Lund, *op. cit.* (note 25), p. 57.

27 John Locke, quoted in Lund, *op. cit.* (note 25), p. 61.

28 Hume, *op. cit.* (note 19), p. 21.

29 Wellcome Library, MS/1250 (Charles Blagden, notebook on memory, undated).

30 Royal Society, CB/3/3, f. 26v (Charles Blagden, diary entries, 26 and 29 Oct. 1794).

31 Royal Society, CB/3/4, f. 5v (Charles Blagden, diary entry, 27 Mar. 1802).

32 British Library, Add MS 33272, f. 172r (letter from Charles Blagden to Joseph Banks, 1 Apr. 1802).

33 Hume, *op. cit.* (note 19), p. 22.

34 Royal Society, CB/4/7/20 (draft of letter from Charles Blagden to William Cullen, undated).

35 Royal Society, CB/1/5/117 (draft of letter from Charles Blagden to Thomas Blagden and Mrs Nelmes, early 1770s); Royal Society, CB/1/3/113 (letter from Thomas Curtis to Charles Blagden, 7 Jan. 1770).

36 Royal Society, CB/1/6/102 (letter from Rev. J. Smart to Charles Blagden, 22 Sep. 1769).

37 David Philip Miller, ‘Blagden, Sir Charles (bap. 1748, d. 1820)’, in *Oxford Dictionary of National Biography* (Oxford University Press, 2004); Royal Society, CB/1/5/113 (draft of letter from Charles Blagden to Mrs Nelmes, 1775).

38 Matthew D. Eddy, ‘The interactive notebook: how students learned to keep notes during the Scottish Enlightenment’, *Book Hist.* **19**, 86–131 (2016), at 86, 121.

39 See Sylas Neville, *The Diary of Sylas Neville 1767–1788* (ed. Basil Cozens-Hardy) (Oxford University Press, London, 1950). For mention of Benjamin Rush's Scottish journal, see Catherine Jones, ‘Benjamin Rush, Edinburgh medicine and the rise of physician autobiography’, in *Scottish Medicine and Literary Culture, 1726–1832* (ed. Megan J. Coyer and David E. Shuttleton) pp. 97–122 (Rodopi, Amsterdam and New York, 2014), at p. 101.

40 Julia Epstein, *Altered conditions: disease, medicine, and storytelling* (Routledge, New York, 1995), pp. 36–40.

41 *Ibid.*, p. 25.

42 See, for example, Wellcome Library, MS/1235 (Charles Blagden, ‘Clinical reports’, 1767).

43 Royal Society, CB/4/5, folder BLA.5.4 (Charles Blagden, notes on patients and symptoms).

44 When Blagden returned from America in 1780, he was initially stationed in the military hospital at Plymouth. After two years, Blagden moved to London, and undertook what Jungnickel and McCormmach have described as a ‘change of career’, in the first instance becoming assistant to Henry Cavendish, whilst forging closer ties with scientific communities in London, and the Royal Society. See Jungnickel and McCormmach, *op. cit.* (note 15), pp. 293–295.

45 Royal Society, CB/4/5, folder BLA.5.2 (Charles Blagden, notes on patients and symptoms, case of Robert Watts, Dec. 1782).

46 David Allan, *Commonplace books and reading in Georgian England* (Cambridge University Press, 2010), pp. 35–36.

47 *Ibid.*, pp. 39–40.

48 Ann Moss, *Printed commonplace-books and the structuring of Renaissance thought* (Clarendon Press, Oxford, 1996), p. 147.

49 Allan, *op. cit.* (note 46), pp. 20, 34.

50 See, for example, Blagden's entry dated 23 September 1771, in which he details a visit made to the blind organist Thomas Grenville. Wellcome Library, MS/1245 (Charles Blagden, commonplace book, entry 23 Sep. 1771).

51 Benjamin Rush's commonplace books are published in Rush, *op. cit.* (note 22); see p. 171.

52 Yeo, *op. cit.* (note 11), p. 187.

53 Francis Bacon, quoted in Blair, *op. cit.* (note 10), p. 69.

54 Patrick J. Murphy and Fred Porcheddu, ‘The antiquarian diaries of Thomas Hearne and Mr Poynter in the fiction of M. R. James: duty unfulfilled’, *Engl. Lit. Transition* **55**, 339–360 (2012), at 342.

55 Elizabeth Yale, ‘Marginalia, commonplaces, and correspondence: scribal exchange in early modern science’, *Stud. Hist. Phil. Biol. Biomed. Sci.* **42**, 193–202 (2011), at p. 195.

56 Thomas Hearne, *Remarks and collections of Thomas Hearne* (ed. Charles Edward Doble) (Clarendon Press, Oxford, 1885), vol. 1, p. 3.

57 Wellcome Library, MS/1245 (Charles Blagden, commonplace book, entry 11 Jan. 1770).

58 John Gascoigne, ‘Banks, Sir Joseph, baronet (1743–1820), naturalist and patron of science’, in *Oxford Dictionary of National Biography* (Oxford University Press, 2004).

59 Letter from Joseph Banks to Charles Blagden, 7 Apr. 1790 (letter 985), published in Neil Chambers (ed.), *The Scientific Correspondence of Sir Joseph Banks, 1765–1820*, 6 vols (Pickering & Chatto, London, 2007), vol. 3, p. 548.

60 Blagden considered himself as pursuing a more ‘literary line’ of work during his association with Banks. See British Library, Add MS 33272, f. 81r–v (letter from Blagden to Banks, 8 Apr. 1790).

61 Beinecke Library, OSB MSS 51, box 1 folders 12 and 14 (Charles Blagden, miscellaneous geographical notes and extracts, undated).

62 Beinecke Library, OSB MSS 51, box 1 folder 12 (Charles Blagden, extract from Pennant, in miscellaneous geographical notes, undated).

63 Beinecke Library, Osborn fc16, box 1 folder 1 (Charles Blagden, diary entry, 23 Jan. 1776).

64 *Ibid*.

65 James Kelly, ‘Bordering on fact in eighteenth-century sea journals’, in *Recording and reordering: essays on the seventeenth- and eighteenth-century diary and journal* (ed. Dan Doll and Jessica Munns), pp. 158–184 (Bucknell University Press, Lewisburg, PA, 2006), at pp. 159–166.

66 Beinecke Library, Osborn fc16, box 1 folder 5 (Charles Blagden, diary entry, 19 Feb. 1777).

67 Henderson, *op. cit.* (note 9), p. 130.

68 Yeo, *op. cit.* (note 11), pp. 190–195, 228.

69 Jan Golinski, *British weather and the climate of enlightenment* (University of Chicago Press, 2007), pp. 137–138.

70 Beinecke Library, Osborn fc16, box 2 folder 15 (Charles Blagden, diary entry, 10 Jun. 1780).

71 See, for example, Blagden's notes on Lapland and Iceland, extracted from a number of sources, in Beinecke Library, OSB MSS 51, box 1 folder 10 (Charles Blagden, notes on Lapland and Iceland, undated).

72 Beinecke Library, Osborn fc16, box 1 folder 1 (Charles Blagden, diary entry, 23 Jan. 1776).

73 Warren Dawson's calendar of Banks's manuscript correspondence in Britain reveals that, although Blagden and Banks were in correspondence from around 1773, the frequency of their letter exchange dramatically increased from 1776, as Blagden sailed for America. See Warren R. Dawson, *The Banks letters: a calendar of the manuscript correspondence of Sir Joseph Banks, preserved in the British Museum, the British Museum (Natural History) and other collections in Great Britain* (Trustees of the British Museum, London, 1958), pp. 50–53.

74 See, for example, letter from Charles Blagden to Joseph Banks, 1776 (letter 75), published in Chambers, *op. cit.* (note 59), vol. 1, p. 85.

75 James Delbourgo, ‘Collecting Hans Sloane’, in *From books to bezoars: Sir Hans Sloane and his collections* (ed. Alison Walker, Arthur MacGregor and Michael Hunter), pp. 9–23 (British Library, London, 2012), at p. 16.

76 *Ibid.*, pp. 16–18.

77 Royal Society, CB/1/1/81 and CB/1/1/82 (draft of letter from Blagden to Banks, 19 Jul. 1782, and Banks's reply, 19 Aug. 1782).

78 See Reginald Heber Howe, ‘Sir Charles Blagden, earliest of Rhode Island ornithologists’, *Am. Nat.* **39**, 397–404 (1905); Natural History Museum, Zoology Manuscripts MSS MCA (transcript of letters from Blagden to Banks, 12 Sep. 1778 and 28 Oct. 1777).

79 Natural History Museum, Zoology Manuscripts MSS MCA (transcript of letter from Charles Blagden to Joseph Banks, 28 Oct. 1777).

80 Beinecke Library, Osborn fc16, box 1 folder 5 (Charles Blagden, diary entry, 18 Feb. 1777).

81 Beinecke Library, OSB MSS 51, box 2 folder 24 (Charles Blagden, calendar of birds, fish & other wildlife, 1777).

82 Natural History Museum, Zoology Manuscripts MSS MCA (transcript of letter from Charles Blagden to Joseph Banks, 12 Sep. 1778).

83 Blagden was particularly supportive of Banks at the Royal Society during the ‘dissensions’ of 1783–1784. See John Gascoigne, *Joseph Banks and the English Enlightenment: useful knowledge and polite culture* (Cambridge University Press, 1994), p. 11.

84 Royal Society, CB/3/3, f. 49r. (Charles Blagden, diary entry, 3 Mar. 1795).

85 Blagden detailed the circumstances of del Campo's promotion to ambassador in his diary: see Royal Society, CB/3/3, f. 48r (Charles Blagden, diary entry, 25 Feb. 1795).

86 In an earlier diary entry, Blagden recorded that his brother was to forward the money from the legacy to arrive on 2 March. Reference to the death of Blagden's uncle in the spring of 1794, and mention of the money that Blagden was to receive in the will, can be found in letters between him and his brother John between May and August 1794, suggesting that the sum mentioned in the diary may have been the legacy from Blagden's uncle. See Royal Society, CB/3/3, f. 47v (Charles Blagden, diary entry 23 Feb. 1795); Beinecke Library, OSB MSS 51, box 5 folder 60 (letters from Charles Blagden to John Blagden Hale, 14 May–13 Aug. 1794).

87 Kenneth Garlick and Angus Macintyre, ‘Introduction’, in *The diary of Joseph Farington* (ed. Kenneth Garlick and Angus Macintyre), pp. xi–xxxix (Yale University Press, New Haven and London, 1978), at pp. xi–xix.

88 Farington, *op. cit.* (note 13), p. xxxiii.

89 *Ibid*., p. 6.

90 Henry Lyons, *The Royal Society 1660–1940: a history of its administration under its charters* (Cambridge University Press, 1944), p. 50.

91 See, for example, Royal Society, CB/3/3, f. 69v (Charles Blagden, diary entry 11 Oct. 1795). This entry mentions Blagden's correspondence with the Swiss mathematician Simon Antoine Jean L'Huilier concerning his paper publishing the following year, entitled ‘Manière élèmentaire d'obte suites par lesquelles s'expriment les quantités exponentielles et les fonctions-gonomètriques des arcs circulaires’, *Phil. Trans. R. Soc. Lond.* **86**, 142–165 (1796).

92 Royal Society, CB/3/3, f. 70v (Charles Blagden, diary entry, 19 Oct. 1795).

93 Royal Society, CB/3/3, f. 77v (Charles Blagden, diary entry, 19 Nov. 1795).

94 T. E. Allibone, ‘The Thursday's Club called the Club of the Royal Philosophers, and its relation to the Royal Society Club’, *Notes Rec. R. Soc.* **26**, 73–80 (1971), at 73.

95 Lyons, *op. cit.* (note 90), p. 74.

96 Steven Shapin, ‘The invisible technician’, *Am. Sci.* **77**, 554–563 (1989), at 560–561.

97 Royal Society, CB/1/1/112 (draft letter from Blagden to Banks, 2 Feb. 1788).

98 British Library, Add MS 33272, f. 78r. (letter from Blagden to Banks, 3 Apr. 1790).

99 Royal Society, CB/1/1/120 (letter from Banks to Blagden, 27 Apr. 1797).

100 Letters held at the Royal Society indicate that Blagden and Elizabeth Grey corresponded over at least a 30-year period between 1784 and 1814: see Royal Society, CB/1/4/76–85. Charles Blagden and Lady Palmerston exchanged a number of letters between 1797 and 1804; for Blagden's letters, see Beinecke Library, Osborn c114.

101 See Royal Society, CB/1/4/76–85 (letters from Elizabeth Grey to Charles Blagden, 8 Jun. 1784–29 May 1814).

102 Royal Society, CB/1/3/277 (letter from Georgiana Cavendish to Blagden, 18 Nov. 1793); Royal Society, MS/821 (Charles Blagden, diary entry, 22 Nov. 1793).

103 Elizabeth Eger, *Bluestockings: women of reason from Enlightenment to Romanticism* (Palgrave Macmillan, Basingstoke and New York, 2010), pp. 60–61.

104 Royal Society, CB/3/3, f. 63r (Charles Blagden, diary entry, 24 Jun. 1795).

105 Hannah More, quoted in J. G. Crowther, *Scientists of the Industrial Revolution* (Cresset Press, London, 1962), p. 333.

106 Royal Society, CB/3/3, f. 53r (Charles Blagden, diary entry, 3 Apr. 1795).

107 In 1795, Blagden assisted Banks with his work for the Privy Council on determining the methods of manufacture of saltpetre in France, as recorded in the diary. See Royal Society, CB/3/3, f. 46r (Charles Blagden, diary entry, 11 Feb. 1795).

108 Royal Society, CB/3/3, ff. 40v–41v (Charles Blagden, diary entries, 1, 2 and 8 Jan. 1795).

109 Henderson, *op. cit.* (note 9), pp. 130–131.

110 Elaine Leong, ‘Collecting knowledge for the family: recipes, gender and practical knowledge in the early modern English household’, *Centaurus* **55**, 81–103 (2013), at 81.

111 For Banks's networks of patrons and clients, see John Gascoigne, *Science in the service of empire: Joseph Banks, the British state and the uses of science in the age of revolution* (Cambridge University Press, 1998). For the patronage networks of Hans Sloane, see Walker, MacGregor and Hunter, *op. cit.* (note 75).

112 On the concept of sociomateriality, see Lissa Roberts and Simon Werrett, ‘Introduction: a more intimate acquaintance’, in *Compound histories: materials, governance, and production, 1760–1840* (ed. Lissa Roberts and Simon Werrett), pp. 1–33 (Brill, Leiden, 2017).

